# Cook County Medical Society

**Published:** 1856-01

**Authors:** 


### EDITORIAL.

*Cook County Medical Society.*

The regular monthly meeting of this society was held on the first Wednesday evening in the month, and was well attended. In addition to other matters of interest which were presented during the evening, Dr. H. A. Johnson reported the following case:

Mr. B. . . aged about 50 years, had been subjected for a considerable time to symptoms of indigestion, and in the early part of last summer, the attention of his physician, Dr. W. B. Herrick, was directed to the secretion of the kidneys. On examination, it was found that he made a *larger* quantity of urine than natural; that it was of low specific gravity, and contained considerable albumen. During the months of September and October, the Urine became much diminished in quantity, but it still contained albumen, and the symptoms of indigestion were much increased, accompanied by frequent vomiting, and often distressing palpitations of the heart.

The vomiting did not always depend on the taking of food or drink, but would recur without either, the matter ejected being chiefly a watery or serous fluid. All these symptoms continued without abatement, coupled with much nervous excitability, until sometime in the month of November, when the vomiting ceased and was speedily followed by delirium and spasmodic action in the muscles of voluntary motion. The delirium was accompanied by so much nervous excitement and disturbance of the heart's action, that a dose of morphine was administered, which lessened the excitement and produced some sleep. The bowels were then freely moved by cathartic medicine, but symptoms of coma began to be exhibited, together with decided spasmodic action in the muscles of the lower extremities. This spasmodic action extended regularly from below upward, until it involved nearly the whole muscular system, and presented the appearance of general convulsions.

The patient soon became entirely comatose, and died. The *post mortem* examination revealed no morbid appearance of importance, except in the kidneys. These were larger and more flabby than natural; color externally paler, and mottled; the capsules were softened and easily detached from the bodies; and the latter, when laid open, presented a well marked granular appearance, with a few points of pus in the cortical part.

As points of special interest connected with this case, Dr. Johnson called the attention of the Society to the connection between the diminished secretion of urine, during the latter part of summer and autumn, and the daily vomiting; the sudden supervention of delirium on the cessation of the vomiting, in November, ending in coma; and the severe spasmodic action of the muscular system, commencing in the lower extremities and extending upward over the trunk and superior extremities by a regular progressive advancement. It has long been supposed that when the pathological condition of the kidneys is such that they fail to secrete urea, the latter being retained in the blood, is capable of exciting that grade of irritation in the mucous membranes of the stomach and bowels, which results not only in diarrhœa or vomiting, but also in the elimination of itself with the fluid evacuated, sometimes unchanged and at others in the form of ammonia. It is when the mucous membrane fails to perform this vicarious office, that the urea is retained in the blood in sufficient quantities to affect the brain, inducing convulsions or coma, or both. Dr. Johnson stated that some of the fluid ejected from the stomach, while the vomiting was frequent and troublesome, was retained for analysis. It emitted an ammonial odor and re-action, but the length of time which had elapsed before the examination was made, rendered it doubtful whether the presence of ammonia was not due to decomposion after having been ejected from the stomach.

In answer to questions from other members of the Society, Dr. Johnson stated that he did not consider the presence of albumen in the urine, as sufficient, of itself, to justify the conclusion that the kidneys had undergone that granular degeneration which has been styled *albumanura*, or Bright's disease. But when, in addition to the presence of more or less albumen, the microscope reveals the presence also of *fat* granules and epithelial cells, there can be little or no room for doubt in regard to the diagnosis.

Dr. S. N. Davis, related a case, then existing in the Mercy Hospital, which strikingly illustrated the effects of retention of urea and other elements of urine, on the mucous membranes of the alimentary canal and the brain. The patient was a native of Ireland, aged about 50 years; and when brought to the Hospital, two months since, presented all the symptoms of anemia, coupled with extensive dropsical effusions into the cellular tissue of the lower extremities and lower part of the trunk, and also moderately into the abdominal cavity. His pulse was small—100 per minute, and soft; surface of the tongue red, clean, and fissured; a sense of burning or heat in the abdomen, increased by food or drink; vomiting occasionally, and from three to six thin serous evacuations from the bowels every day, sometimes accompanied by griping and tenesmus. The quantity of urine secreted was very small, not exceeding two or three ounces in the twenty-four hours, pale and nearly odorless. On subjecting it to the action of heat and nitric acid, the albuminous precipitate was so copious as to render the whole mass solid. According to the representations of the patient, he had been attacked with fever, in St. Louis, about two months previous, from which he had only partially recovered, when he began to feel an unusual pain in the region of the kidneys, speedily followed by scantiness of urine and the commencement of dropsical effusions into the cellular tissues of the lower extremities and abdomen. In eight or ten days after the first appearance of dropsical swellings, the serous diarrhœa commenced, which has continued, with only brief intervals, until the present time. The patient has now been in the Hospital two months, and it has uniformly happened, that whenever the discharges from the bowels were suppressed for twenty-four hours, he first became very restless, and then manifiested signs of approaching coma. About four weeks since, the skin on the back part of the legs and thighs became first inflamed, and then excoriated; and from these began to flow considerable of the water effused into the cellular tissue. In one week nearly all the water effused into the cellular tissue of the trunk and extremities, had passed off through these excoriated surfaces. But no improvement took place in the secretory action of the kidneys—during some days the quantity of urine discharged being less than a single fluid ounce. In this case, the uniformity with which the brain and nervous system became disturbed, when the diarrhœa was checked, together with the exceedingly small quantity of urine which has been secreted during several successive weeks, leaves no doubt but that the action of the mucous membrane of the intestines, constitutes a substitute, in some degree at least, for that of the kidneys. If this view is correct, it is evident that no attempt should be made by the physician to arrest the diarrhœa, without first securing the establishment of a more copious and healthy secretion of urine.

The foregoing cases led to an interesting discussion, in which most of the members present participated more or less; but of their remarks we have not sufficient notes to make a reliable report.

